# Oleic Acid Increases Lipid Accumulation in Duck Hepatocytes by Promoting Apolipoprotein A1 Expression

**DOI:** 10.3390/ani15243603

**Published:** 2025-12-15

**Authors:** Ziyi Pan, Xuewen Li, Dongsheng Wu, Longfei Xie, Xingyong Chen, Zhaoyu Geng

**Affiliations:** 1 College of Animal Science and Technology, Anhui Agricultural University, Hefei 230036, China; panziyi718@163.com (Z.P.); a15936572117@163.com (X.L.); a18437969621@163.com (D.W.); 13188265421@163.com (L.X.); chenxingyong@ahau.edu.cn (X.C.); 2 Tropical Crops Genetic Resources Institute, Chinese Academy of Tropical Agricultural Sciences, Haikou 571101, China; 3 Nanyang Agriculture and Rural Affairs Bureau, Nanyang Animal Husbandry Development Center, Nanyang 473399, China

**Keywords:** duck, hepatocyte, oleic acid, APOA1, lipid accumulation

## Abstract

Background: Lipid overaccumulation in the liver predisposes ducks to metabolic disorders. The molecular mechanism of oleic acid (OA)-induced hepatic steatosis in ducks is not fully elucidated. Methods: A cellular model of steatosis was established by treating primary duck hepatocytes with OA. Transcriptome sequencing was performed to identify key signaling pathways and candidate genes. The role of Apolipoprotein A1 (APOA1) was investigated through overexpression and knockdown experiments. Intracellular triglycerides (TGs) were quantified commercially; lipid droplets were visualized by Oil Red O staining. Results: Intracellular TG accumulation was induced by OA treatment in a dose-dependent manner. Through transcriptome analysis, 1045 differentially expressed genes (DEGs) were identified, with APOA1 being recognized as a key candidate within the peroxisome proliferator-activated receptor (PPAR) signaling pathway. The content of TGs and lipid droplets was increased by APOA1 overexpression, whereas these effects were suppressed by APOA1 knockdown. The expression of acetyl-CoA carboxylase alpha (ACACA) and fatty acid synthase (FASN) was upregulated by APOA1. Conversely, the expression of carnitine O-palmitoyltransferase 1 (CPT1), acyl-CoA oxidase 1 (ACOX1), and apolipoprotein B (APOB) was downregulated. Conclusions: This study demonstrates that OA upregulates APOA1, suggesting the involvement of the PPAR pathway and providing a theoretical basis for modulating hepatic fat deposition.

## Simple Summary

While capable of physiological fat storage, the duck liver is susceptible to metabolic disorders. This study explored the mechanism of oleic acid (OA)-induced fat accumulation in duck hepatocytes. The results of this study showed that OA activates the PPAR signaling pathway, leading to increased expression of APOA1. This upregulation enhances intracellular lipid synthesis while simultaneously inhibiting lipid degradation and transport, ultimately leading to hepatic steatosis.

## 1. Introduction

The duck liver is a central organ for lipid metabolism. Hepatic steatosis, characterized by lipid accumulation, can occur spontaneously as a physiological process for energy storage or be induced by short-term overfeeding with a high-energy diet [1]. This process results in significant liver enlargement, which is the basis for “foie gras” production [2]. However, excessive fat deposition can lead to health issues such as steatohepatitis and liver fibrosis [3,4]. The pathogenesis of this condition involves a complex imbalance in lipid homeostasis, including exacerbated de novo lipogenesis, the overexpression of lipogenic genes, accelerated triglyceride synthesis [5], and is often compounded by increased fatty acid uptake, diminished β-oxidation, and reduced very-low-density lipoprotein (VLDL) secretion [6].

Given its close pathological resemblance to human non-alcoholic fatty liver disease (NAFLD), duck hepatic steatosis constitutes a valuable translational model [7,8,9]. For in vitro investigations, the use of primary hepatocytes is favored for its reproducibility and ease of manipulation. In this context, oleic acid (OA), a predominant monounsaturated fatty acid, has been established as a robust and widely utilized agent for inducing steatosis in hepatocytes from chickens and other mammals [5,10]. Nevertheless, the establishment and application of a well-characterized OA-induced model in duck hepatocytes remain limited.

Genetic predisposition is a major factor in duck hepatic steatosis, with significant variation in susceptibility observed across different breeds [11,12]. The underlying metabolic disruptions converge on altered carbohydrate and lipid metabolism, enhancing glycolysis and de novo lipogenesis while reducing the synthesis, secretion, and uptake of apolipoproteins [12,13,14,15]. Apolipoprotein A1 (APOA1), the principal structural and functional component of high-density lipoprotein (HDL), plays a well-established role in reverse cholesterol transport and exhibits anti-inflammatory properties [16,17]. In this process, cholesterol esters are transferred to the liver via the SR-B1 receptor or indirectly via CETP to LDL/VLDL for hepatic uptake by the LDL receptor, ultimately leading to biliary excretion, which is crucial for maintaining hepatic metabolic balance [18]. Notably, the triglyceride-to-APOA1 ratio has recently emerged as a promising biomarker for human hepatic steatosis [19]. However, despite these clinical associations and its central role in lipid transport, the direct impact of APOA1 on triglyceride accumulation and its precise molecular mechanism in duck hepatocytes remain entirely unexplored.

Therefore, this study was designed to systematically investigate the mechanism of OA-induced steatosis in duck hepatocytes. A combined approach of biochemical, transcriptomic, and functional genetic analyses was employed. A robust steatosis model was established using OA-treated primary duck hepatocytes. Transcriptome profiling was then conducted to identify critical pathways and candidate genes, leading to the selection of APOA1 for in-depth functional validation. Our study thus delineates a novel pathway in duck hepatic steatosis and provides a foundational reference for addressing fat metabolism disorders in poultry.

## 2. Materials and Methods

### 2.1. Duck Primary Hepatocyte Isolation and Culture

The animal experiments were conducted in accordance with the Regulations and Guidelines on Animal Management, and the experiments were approved by the Animal Care and Use Committee of Anhui Agricultural Capital Institution (no: SYXK 2016-007).

Primary duck hepatocytes were isolated from 18-day-old Cherry Valley duck embryos [20]. The livers were aseptically dissected, rinsed, minced, and digested with 0.2 mg/mL type IV collagenase at 37 °C for 25 min. The digest was filtered through 300 μm and 200 μm meshes, centrifuged at 800× *g* for 5 min, and treated with red blood cell lysis buffer. Cells were resuspended in complete medium (89% F12/DMEM, 10% FBS, 1% penicillin-streptomycin) and maintained at 37 °C with 5% CO_2_. Cells from each embryo constituted one independent biological replicate. For subsequent experiments, cells from a single embryo were allocated across multiple culture vessels: one portion was seeded into 6-well plates for triglyceride (TG) assay and Oil Red O staining, and another portion into a 96-well plate for cell viability assay. Experiments were repeated using hepatocytes from three independent embryos (*n* = 3). For transcriptome sequencing, cells from three embryos were each split into control and OA-treated groups, yielding 6 samples in total. Functional assays (qPCR, Western blot) also utilized cells from three independent embryos.

### 2.2. Oleic Acid-Induced Steatosis Model Establishment

At approximately 70% confluence, hepatocytes were treated with oleic acid (OA) pre-complexed with fatty acid-free bovine serum albumin (BSA). The OA-BSA complex was diluted in complete medium to final concentrations of 75, 150, and 225 μM. Control cells (0 μM) received a vehicle solution containing 2% (*w*/*v*) fatty acid-free BSA in complete medium. Cells were harvested for analysis after 24, 48, 72, and 96 h of treatment.

### 2.3. Cytotoxicity and Triglyceride Quantification

Cell viability and intracellular triglyceride levels were assessed with commercial kits. The CCK-8 assay (A311, Vazyme, Nanjing, China) was used for viability, with absorbance read at 450 nm after a 2 h incubation. For triglyceride (TG) content, an enzymatic kit (E1013, Applygen, Beijing, China) was employed, and the measured values were normalized to the total protein concentration as determined by a BCA assay (E112, Vazyme, Nanjing, China). A microplate reader was used for all absorbance measurements. Each experiment included three biological replicates.

### 2.4. Oil Red O Staining

Fat staining and quantification refer to Pan et al. [21]. In brief, cells were stained with a filtered Oil Red O working solution (prepared from a 0.5% stock in isopropanol, diluted 6:4 with water), and lipid accumulation was quantified as described.

Following treatment, cells were washed with phosphate-buffered saline (PBS), fixed with 4% paraformaldehyde for 30 min, and then incubated with the filtered working solution for 30 min. After staining, cells were thoroughly rinsed with distilled water to remove excess dye. The stained lipid droplets were imaged under a light microscope. The images were acquired by the researcher using a DP73 microscope with a 20× objective. For quantification, the incorporated dye was eluted with 100% isopropanol and the absorbance was measured at 510 nm.

### 2.5. Transcriptome Sequencing and Bioinformatic Analysis

Total RNA was isolated from 6 samples using TRIzol^®^ (Invitrogen, CA, USA) and reverse-transcribed to cDNA (1 μg RNA; Vazmay kit, Nanjing, China). RNA quality was assessed via Bioanalyzer 2100 with RNA 6000 Nano Kit (Agilent, CA, USA), selecting samples with OD260/280 = 1.9–2.0. The transcriptome sequencing and analysis were conducted by OE Biotech Co., Ltd. (Shanghai, China). Raw reads were quality-trimmed (Trimmomatic, München, Bavaria, Germany) and aligned to the Pekin duck genome (GCA_003850225.1) via TopHat. Gene quantification was done with HTSeq, and differential expression was analyzed using DESeq2 v.1.48.2, with significant differentially expressed genes (DEGs) defined as |log_2_FC| > 1.5 and adjusted *p* < 0.05. Functional enrichment of DEGs was performed for the Gene Ontology (GO) terms and the Kyoto Encyclopedia of Genes and Genomes (KEGG) pathways (significance threshold: corrected *p* < 0.05).

### 2.6. Overexpression and Knockdown of APOA1 Gene in Duck Primary Hepatocytes

For APOA1 overexpression, the complete coding sequence (CDS) of duck APOA1 (XM_027444321) was cloned into the pBI-CMV3 vector (Pharma, China) using the NheI and HindIII restriction sites. The specific shRNA (5′-CCGGGGACAGGTCTTCAGGTAAACACTCGAGTGTTTACCTGAAGACCTGTCCTTTTTG-3′) of APOA1 was designed and synthesized by Pharma Biotechnology (Shanghai, China). The constructed vectors were transfected into primary duck hepatocytes using ExFect Transfection Reagent (Vazyme, T101, Nanjing, China) according to the manufacturer’s protocol, with a reagent-to-vector ratio of 2:1. The control groups for APOA1 overexpression and knockdown were the overexpression normal control (oeNC) and the knockdown normal control (shNC), respectively. Transfection efficiency was confirmed by observing green fluorescent protein (GFP) signal using a fluorescence microscope (DP73, Olympus, Hachioji, Japan) equipped with a 4× objective lens at 24 h post-transfection. Cells were harvested for subsequent analysis 24 h post-transfection.

### 2.7. Quantitative Real-Time PCR (qPCR)

Total RNA was extracted from duck hepatocytes using TRIzol reagent (Invitrogen, CA, USA) according to the manufacturer’s instructions. The concentration and purity of the RNA were determined spectrophotometrically. Subsequently, 1 μg of total RNA from each sample was reverse-transcribed into complementary DNA (cDNA) using a commercial RT-cDNA synthesis kit. Quantitative PCR was performed on an ABI7500 Real-Time PCR System (Thermo Fisher Scientific, CA, USA) using a commercial qPCR mix (Q711, Vazyme, Nanjing, China). The reaction system was: 95 °C for 5 min, 95 °C for 30 s, 60 °C for 30 s, 35 cycles. With GAPDH as the reference gene, RT–PCR was used to detect the mRNA levels. The relative mRNA expression levels of the target genes were calculated using the comparative 2^(–ΔΔCt)^ method. The primers were listed in Table S1.

### 2.8. Western Blot

Cells were harvested 24 h post-transfection and lysed in RIPA buffer supplemented with protease inhibitors (Meilunbio, MA0151, Dalian, China) on ice. The lysates were clarified by centrifugation at 12,000× *g* for 10 min at 4 °C, and the supernatant was collected. The protein concentration was determined using a BCA protein assay kit (Vazyme, E112-02). Equal amounts of total protein from each sample were separated by SDS-PAGE and subsequently transferred onto a PVDF membrane (Thermo Fisher, 88518) using a gel-to-membrane transfer system (Bio-Rad, PowerPac™ HV, Hercules, CA, USA). The membrane was then blocked with 5% BSA in TBST (Sangon Biotech, B040126, Shanghai, China) for 2 h at room temperature. Following blocking, the membrane was incubated overnight at 4 °C with primary antibodies against APOA1 (ImmunoWay, YN0018, Beijing, China) and GAPDH (ImmunoWay, YM3215, Beijing, China), diluted in 1% BSA. After washing with TBST, the membrane was incubated with a horseradish peroxidase (HRP)-conjugated secondary antibody (ImmunoWay, Beijing, China) for 2 h at room temperature. Protein bands were visualized using an enhanced chemiluminescence (ECL) kit (Vazyme, E412-01, Nanjing, China) and imaged. The relative band intensities were quantified using ImageJ software 1.50d (Bethesda, Rockville, MD, USA).

### 2.9. Statistical Analysis

Data are presented as the mean ± SD. The experimental unit was defined as primary hepatocytes isolated from a single duck embryo, representing one independent biological replicate. All experiments were performed with n = 3 independent biological replicates. For multi-group comparisons, data were first analyzed by ANOVA. If the ANOVA indicated a significant overall effect, Dunnett’s post hoc test was then applied. ^a,b,c^ Significant differences are shown in superscript letters, with different letters representing significant differences (*p* < 0.05).

## 3. Results

### 3.1. Oleic Acid Induces Lipid Accumulation in Duck Hepatocytes

**Figure 1:**
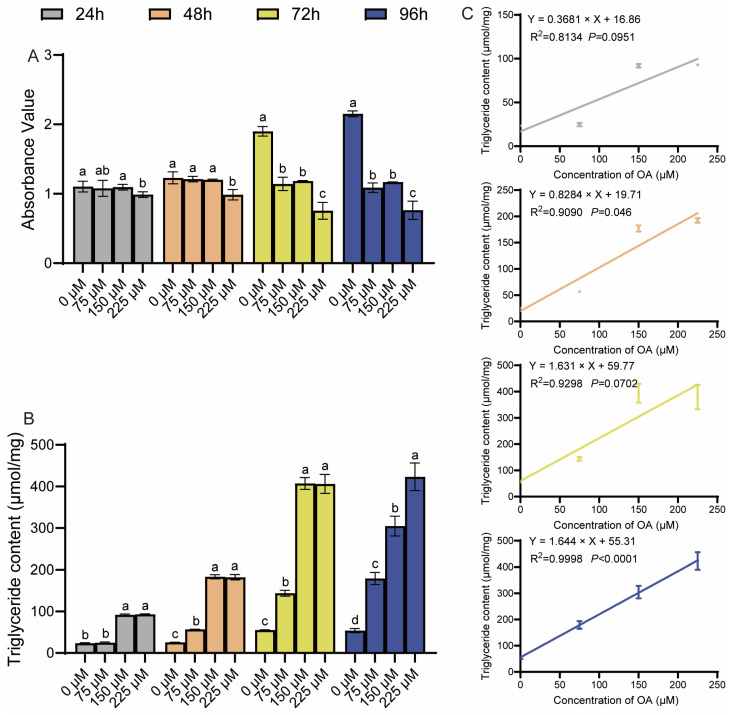
Nontoxic induction of steatosis by oleic acid in duck hepatocytes. (**A**) Cell viability (CCK-8 assay) after OA treatment. (**B**) Intracellular triglyceride (TG) levels. The data are reported as the mean ± SD on the basis of n = 3 independent biological replicates (cells from distinct embryos). ^a,b,c,d^ Significant differences are shown in superscript letters, with different letters representing significant differences (*p* < 0.05). (**C**) Linear correlation between OA concentration and TG content. R^2^ and *p*-values are shown.

To evaluate oleic acid (OA) cytotoxicity, primary duck hepatocytes were treated with various concentrations for different durations. Treatment with 75 and 150 μM OA for up to 48 h did not significantly affect cell viability, whereas 225 μM OA markedly reduced it (Figure 1A). In contrast, a marked reduction in cell viability was observed at 225 μM OA, indicating a toxic effect at this concentration. Concomitantly, intracellular triglyceride (TG) levels were significantly increased by OA treatment in a dose- and time-dependent manner compared to the control (Figure 1B). The relationship between OA concentration and intracellular TG accumulation was further examined by linear regression analysis. Linear regression analysis revealed a strong positive correlation between OA concentration and TG accumulation at 24, 48, and 96 h, but not at 72 h (Figure 1C). Based on these results, concentrations of 75 µM and 150 µM were chosen for all subsequent experiments as they effectively induced hepatic steatosis within a non-cytotoxic range, thereby establishing a valid in vitro model.

### 3.2. Oleic Acid Promotes Lipid Droplet Accumulation in Duck Hepatocytes

**Figure 2:**
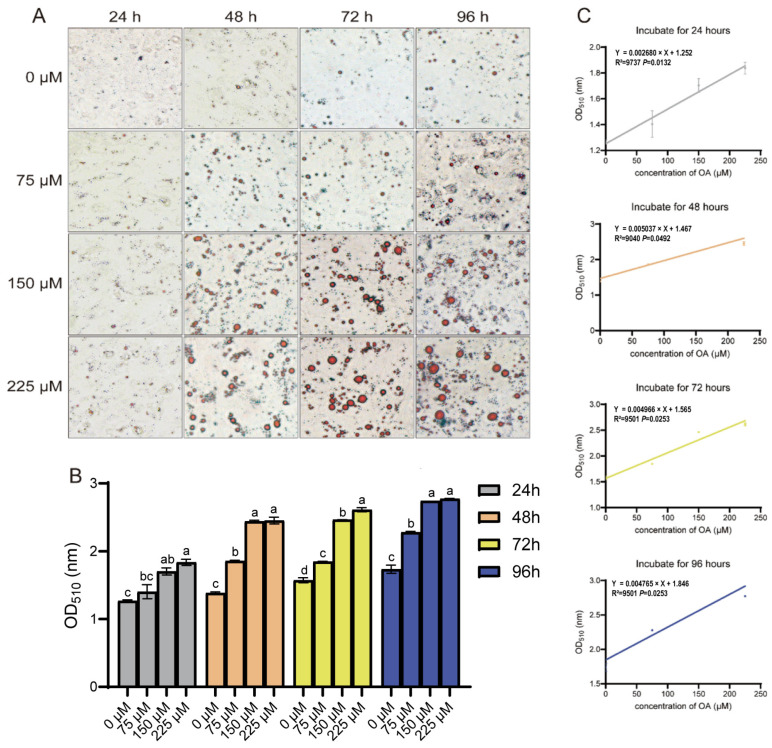
OA concentration-dependent lipid accumulation in duck hepatocytes. (**A**) Oil Red O staining of lipid droplets after OA treatment. Objective lens magnification: 20×. (**B**) Quantification of lipid accumulation from (**A**). The data are reported as the mean ± SD on the basis of n = 3 independent biological replicates (cells from distinct embryos). ^a,b,c^ Significant differences are shown in superscript letters, with different letters representing significant differences (*p* < 0.05). (**C**) Linear correlation between OA concentration and lipid content at indicated times. R^2^ and *p*-values are shown.

The progression of hepatic steatosis was morphologically assessed by Oil Red O staining for lipid droplets. A marked increase in lipid droplet accumulation was observed in OA-treated groups compared with the control (Figure 2A,B). This lipid accumulation was significantly enhanced by higher OA concentrations across the experimental time points, except in hepatocytes treated with 75 μM OA for 24 h. Linear regression analysis confirmed a strong positive correlation between OA concentration and intracellular lipid content at each individual time point (24, 48, 72, and 96 h; Figure 2C). These morphological results are consistent with the TG quantification data, demonstrating that lipid droplet accumulation is effectively induced by OA.

### 3.3. Clustering Analysis of Differentially Expressed Genes

**Figure 3:**
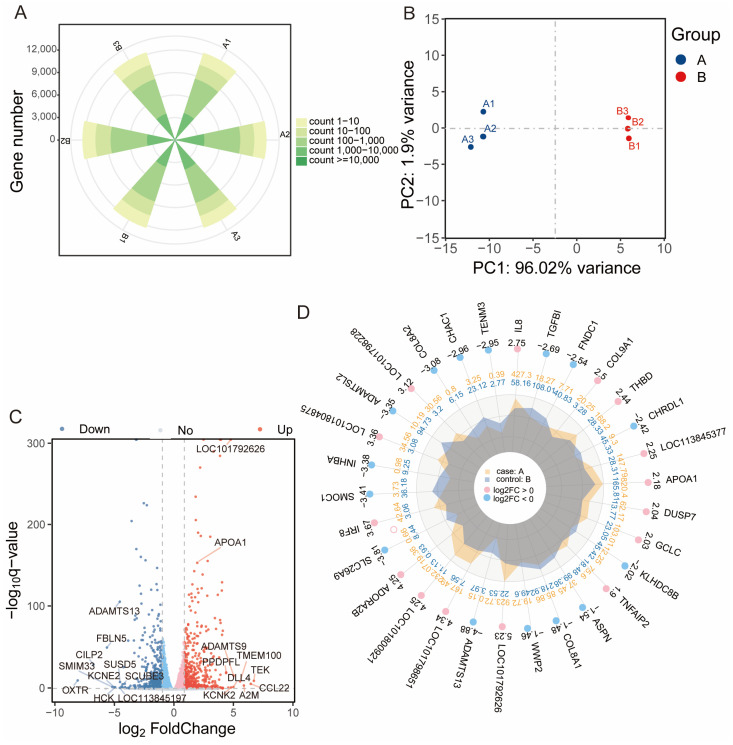
Transcriptomic profiling of OA-treated duck hepatocytes. (**A**) The number of genes detected per sample after quality control and filtering. (**B**) PCA showing separation between control and OA-treated groups. (**C**) Volcano plot of DEGs (adjusted *p* < 0.05, |log_2_FC| > 1.5). (**D**) Radar chart of top 30 DEGs by fold change. A: 150 μM OA cells for 48 h; B: 0 μM OA cells for 48 h.

To investigate the molecular mechanisms underlying OA-induced steatosis, transcriptome sequencing was performed on duck hepatocytes treated with 150 μM OA (group A) and 0 μM OA control cells for 48 h (group B). After quality control and alignment, an average of 12,280 genes were detected per sample (Figure 3A). Principal component analysis (PCA) revealed a clear separation between the A and B groups (Figure 3B). Comparative analysis identified 1045 DEGs, with 523 upregulated and 522 downregulated in group A (Figure 3C and Table S2). The top 30 most significantly upregulated and downregulated genes, ranked by fold change, are presented in Figure 3D.

### 3.4. Functional Enrichment Analysis of DEGs

**Figure 4:**
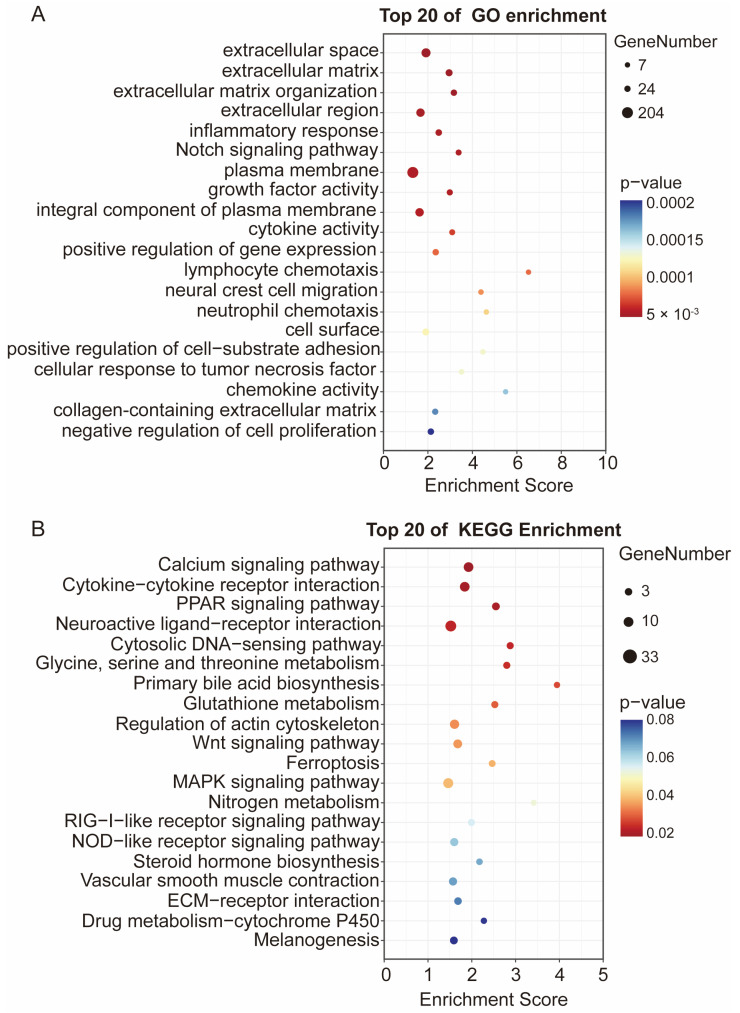
GO and KEGG enrichment analysis of DEGs. (**A**) The GO enrichment of DEGs. (**B**) The KEGG pathway analysis of DEGs.

Functional enrichment analysis was performed to decipher the biological implications of the DEGs. Gene Ontology (GO) analysis revealed that the top 20 enriched terms were predominantly related to extracellular components, such as extracellular space, extracellular matrix, and extracellular matrix organization (Figure 4A). Kyoto Encyclopedia of Genes and Genomes (KEGG) pathway analysis further showed significant enrichment in 20 key pathways, with the peroxisome proliferator-activated receptor (PPAR) signaling pathway, Calcium signaling pathway, and Cytokine-cytokine receptor interaction being the most significantly affected (Figure 4B). The significant enrichment of the lipid metabolism regulator PPAR signaling pathway guided the subsequent focus on its downstream target, apolipoprotein A1 (APOA1).

### 3.5. Candidate Gene Validation and Selection

**Figure 5:**
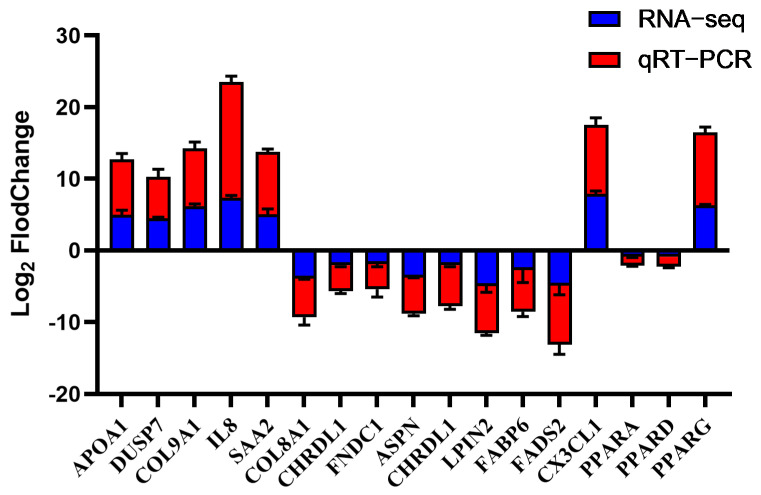
Concordance between qPCR and RNA-seq results for 17 DEGs. The data are reported as the mean ± SD on the basis of n = 3 independent biological replicates (cells from distinct embryos).

To validate the reliability of the transcriptome data, 17 DEGs were selected for qPCR analysis. The results confirmed that their expression trends were consistent with the RNA-seq findings (Figure 5), thereby verifying the accuracy of transcriptome data. Notably, within the PPAR pathway, OA induced a distinct isoform expression profile: PPARG (a lipogenic regulator) was significantly upregulated, whereas PPARA (a fatty acid oxidation master regulator) and PPARD were downregulated. The APOA1 was identified as being associated with the extracellular space from the GO analysis and was also a constituent of the significantly enriched PPAR signaling pathway. Given this dual functional correlation, APOA1 was selected as a key candidate gene for further investigation into its role in hepatic lipid accumulation.

### 3.6. Functional Role of APOA1 in Regulating Triglyceride Accumulation in Duck Hepatocytes

**Figure 6:**
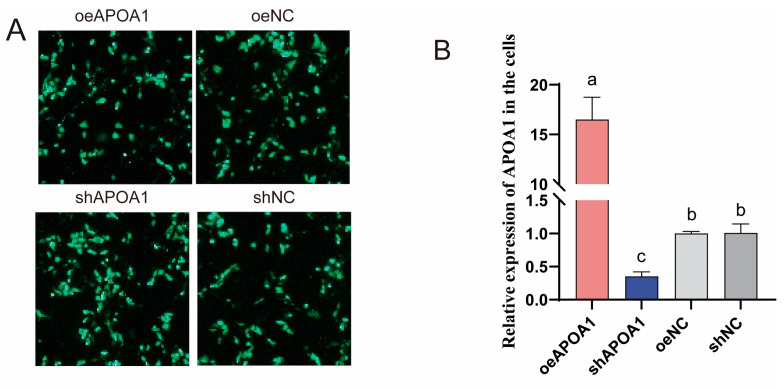

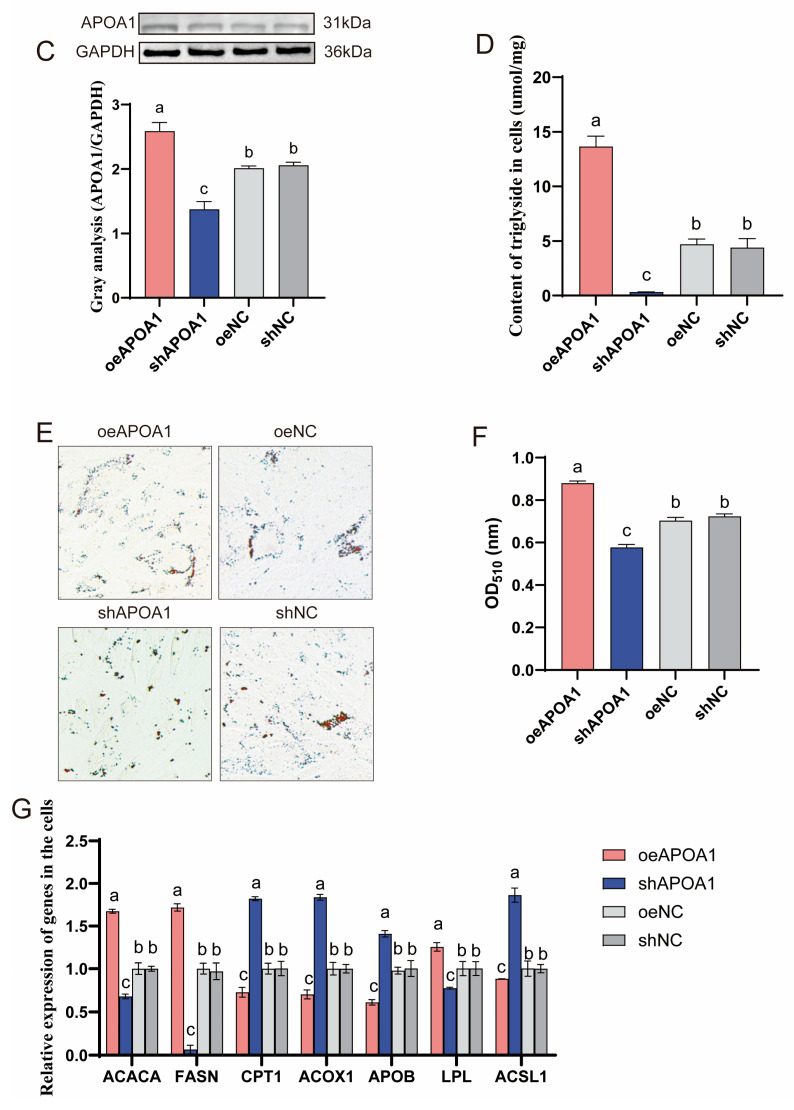
APOA1 regulates triglyceride accumulation in duck hepatocytes by modulating lipid metabolism pathways. (**A**) Fluorescence microscopy images confirming the successful transfection of oeAPOA1, shAPOA1, oeNC and shNC plasmids. Objective lens magnification: 20×. (**B**,**C**) The mRNA (**B**) and protein (**C**) expression levels of APOA1 were validated by qPCR and Western blot, respectively. GAPDH was used as a loading control. (**D**) Intracellular triglyceride (TG) content measured in the duck hepatocytes. (**E**) Representative images of lipid droplets stained with Oil Red O. Objective lens magnification: 20×. (**F**) Quantitative analysis of lipid accumulation from the Oil Red O staining shown in (**E**). (**G**) Expression levels of key lipid metabolism genes. ACACA (Acetyl-CoA carboxylase alpha), de novo lipogenesis; FASN (Fatty acid synthase), de novo lipogenesis; LPL (Lipoprotein lipase), lipoprotein triglyceride hydrolysis; CPT1 (Carnitine palmitoyltransferase 1), rate-limiting step of mitochondrial fatty acid β-oxidation; ACOX1 (Acyl-CoA oxidase 1), rate-limiting step of peroxisomal fatty acid β-oxidation; APOB (Apolipoprotein B), essential structural component of VLDL for lipid export; ACSL1 (Acyl-CoA synthetase long chain family member 1), fatty acid activation. The data are reported as the mean ± SD on the basis of n = 3 independent biological replicates (cells from distinct embryos). ^a,b,c^ Significant differences are shown in superscript letters, with different letters representing significant differences (*p* < 0.05).

To elucidate the functional role of APOA1 in triglyceride metabolism, APOA1 gene overexpression and knockdown studies were conducted in duck hepatocytes. Successful transfection of the APOA1 overexpression (oeAPOA1) and knockdown (shAPOA1) plasmids was confirmed by the presence of green fluorescence (Figure 6A). Subsequent qPCR and Western blot analyses verified a significant increase and decrease in APOA1 expression at the mRNA and protein levels, respectively (Figure 6B,C). Functionally, APOA1 overexpression significantly increased TG levels, whereas its knockdown reduced TG contents (Figure 6D). Consistently, Oil Red O staining demonstrated that oeAPOA1 enhanced lipid droplet accumulation, whereas its shAPOA1 suppressed it (Figure 6E,F). Furthermore, oeAPOA1 promoted the expression of lipogenic genes (ACACA, FASN, LPL) while suppressing genes involved in fatty acid oxidation (CPT1, ACOX1) and lipid transport (APOB, ACSL1). These expression patterns were reversed upon APOA1 knockdown (Figure 6G). These results demonstrate that APOA1 is a key regulator that promotes triglyceride accumulation in duck hepatocytes by coordinately modulating the expression of genes central to lipid synthesis, oxidation, and transport.

## 4. Discussion

Oleic acid (OA), a monounsaturated fatty acid abundant in vegetable oils, can be taken up by hepatocytes and esterified into neutral lipid droplets for storage [5,10,22]. In the present study, a stable and controllable in vitro model of hepatic steatosis was established by treating primary duck hepatocytes with OA at different concentrations and durations. Cytotoxicity assays confirmed that no significant toxicity was induced by treatment with 75 μM or 150 μM OA for 48 h, which ensured that the observed lipid accumulation could be attributed to specific metabolic effects of OA rather than nonspecific cytotoxicity. This finding is consistent with observations reported in chicken hepatocyte lines and mammalian hepatocytes [10,22]. Intracellular TGs and lipid droplets are important indicators of the degree of liver steatosis [5]. Significant lipid accumulation was confirmed at both quantitative and morphological levels by triglyceride content measurement and Oil Red O staining, respectively. Intracellular TG levels and lipid droplet formation were shown to increase in a linear dose-dependent manner with OA concentration. This well-defined quantitative relationship not only provides strong evidence for a direct causal role of OA in steatosis induction but also advances the model from a qualitative observational system to a predictive experimental platform.

Based on this validated model, transcriptome sequencing was performed to investigate the molecular mechanisms underlying OA-promoted triglyceride and lipid accumulation in duck hepatocytes. A total of 1045 DEGs were identified, forming network involved in OA-regulated lipid metabolism. These DEGs were found to be significantly enriched in extracellular space-related terms and the PPAR signaling pathway. PPARs are nuclear receptors known to be activated by fatty acids and their derivatives, and are recognized as key transcription factors in nutritional sensing and lipid metabolism regulation [23,24]. These results suggest that OA functions as a natural ligand that activates the PPAR pathway, leading to altered expression of downstream lipid metabolism-related genes. The results of this study indicate that OA has different regulatory modes for the expression of different PPAR subtypes. The changes in the expression of PPARA/D/G provide a new regulatory mechanism for the metabolic reprogramming of lipid storage in duck hepatocytes. Among these genes, APOA1, a known target of PPAR transcription factors, was identified as a candidate of interest. APOA1 is characterized as the main structural protein of high-density lipoprotein (HDL) and is implicated in intracellular lipid transport and redistribution processes. Although APOA1-mediated reverse cholesterol transport has been traditionally associated with reduced tissue lipid burden [25,26,27], its biological functions have been shown to exhibit considerable complexity and context-dependency across different tissues [26,28]. In this study, OA upregulation of APOA1 occurred in association with PPAR signaling activation and led to enhanced lipid accumulation in hepatocytes. The reason for this difference may be related to the unique physiological environment of duck liver and the specific state induced in this study. The duck liver is a natural high-capacity lipid storage organ. The function of APOA1 in the duck liver may be to act as a key coordinator of intracellular lipid distribution, promoting the safe storage of excess fatty acids into lipid droplets to prevent lipotoxicity. These results indicate that APOA1 may be identified as a key mediator of OA-induced lipid accumulation in duck hepatocytes.

As the primary apolipoprotein of HDL, APOA1 is widely recognized for its role in cholesterol clearance and the alleviation of lipid burden [29,30]. However, in the present study, APOA1 not only stimulates lipid acquisition through upregulating lipogenic genes (ACACA, FASN) [31,32] and inhibits lipid disposal via suppressing β-oxidation (CPT1, ACOX1) [33,34], but also critically impairs lipid export by downregulation of APOB [35]. APOB is a key structural component of very low-density lipoproteins (VLDL) [35]. Overexpression of APOA1 inhibits the expression of the APOB gene, indicating that the lipid efflux function mediated by APOB is restricted, leading to intracellular fat accumulation. These results suggest that APOA1 may increase the risk of hepatocellular steatosis by enhancing lipid synthesis while attenuating lipid breakdown and export. While APOA1 is classically associated with cholesterol efflux, our results suggest that in duck hepatocytes, APOA1 may increase lipid accumulation by reprogramming the expression network of key lipid metabolism genes [36]. Therefore, the primary function of APOA1 in duck hepatocytes is suggested to extend beyond lipid transport, serving as a signal for metabolic reprogramming that ultimately leads to intracellular lipid accumulation. It should also be acknowledged that our study has certain limitations. While we have established APOA1 as a key functional regulator in OA-induced steatosis, the precise transcriptional regulatory relationship between APOA1 and downstream genes such as ACACA, FASN, CPT1, and APOB remains to be fully elucidated. Future studies employing techniques such as chromatin immunoprecipitation (ChIP) or ChIP-sequencing will be crucial to determine whether APOA1 directly binds to the promoters of these genes, thereby delineating the direct transcriptional targets within this metabolic network.

## 5. Conclusions

This study demonstrates that OA induces hepatic steatosis in duck hepatocytes by upregulating APOA1, an effect associated with the activation of the PPAR signaling pathway. APOA1 drives intracellular lipid accumulation by coordinately enhancing lipid synthesis while suppressing fatty acid oxidation and impeding lipid export. The results of this study indicate that APOA1 is a candidate gene regulating lipid metabolism in duck hepatocytes, providing a molecular basis for understanding fat deposition in waterfowl.

## Data Availability

The data related to this paper may be requested from the corresponding author.

## Supplementary Materials

The following supporting information can be downloaded at: https://www.mdpi.com/article/10.3390/ani15243603/s1, Figure S1: Original images of Western blot; Table S1: The primers of genes; Table S2: Differentially expressed genes between Group A and B.
