# Supplementary figures and images for: Oleic Acid Increases Lipid Accumulation in Duck Hepatocytes by Promoting Apolipoprotein A1 Expression
Source: Animals (Basel). 2025 Dec 15;15(24):3603. doi: 10.3390/ani15243603 (PMC12729497; doi:10.3390/ani15243603)

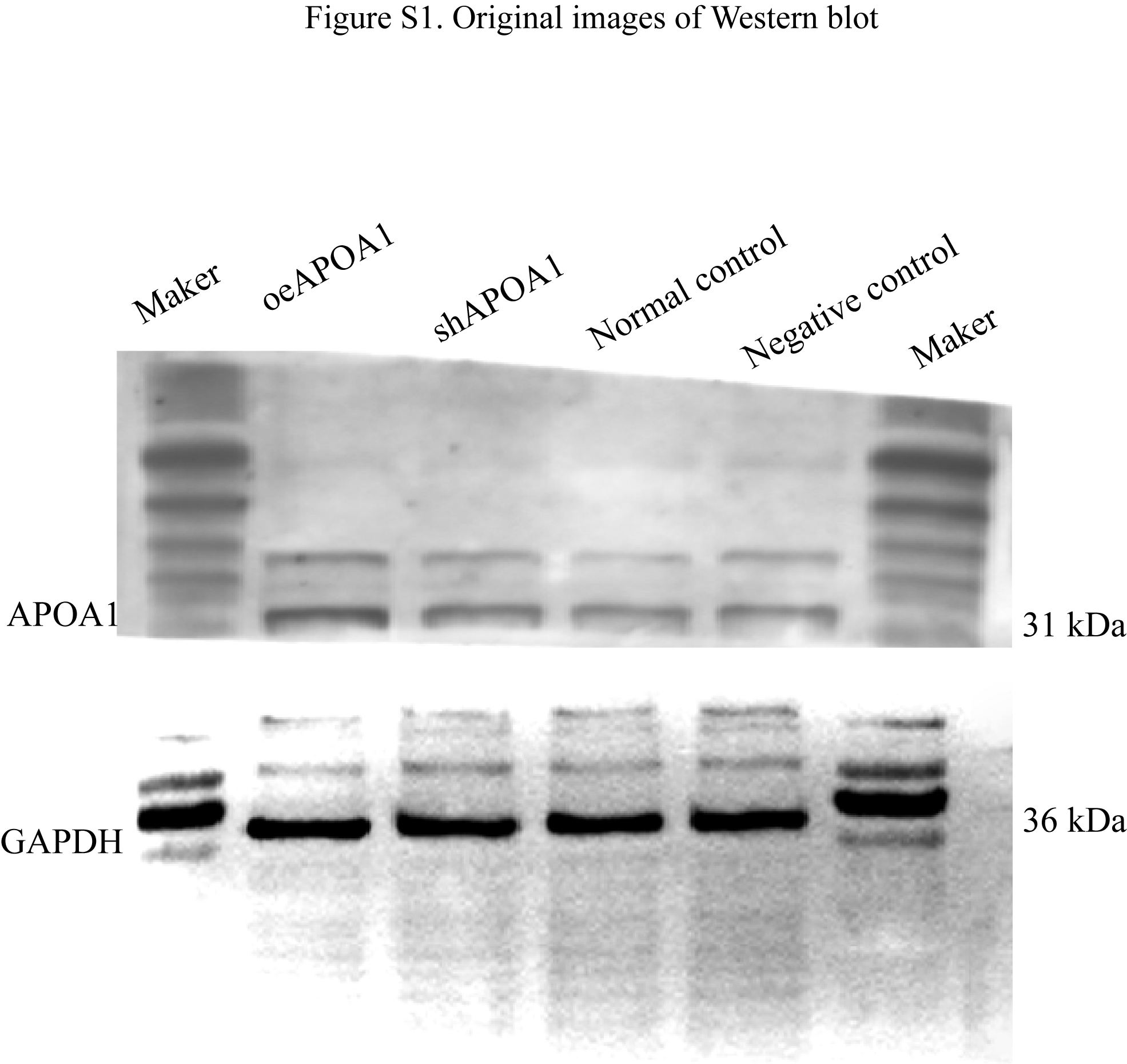

Supplement: Supplementary file 1 [file animals-15-03603-s001.zip › Figure S1. Original images of Western blot.tif]
